# Type 2 Cytokine–Dependent Skin Barrier Regulation in Personalized 2-Dimensional and 3-Dimensional Skin Models of Atopic Dermatitis: A Pilot Study

**DOI:** 10.1016/j.xjidi.2024.100309

**Published:** 2024-08-30

**Authors:** Hila Emmert, Franziska Rademacher, Matthias Hübenthal, Regine Gläser, Hanne Norsgaard, Stephan Weidinger, Jürgen Harder

**Affiliations:** 1 Department of Dermatology, University Hospital Schleswig-Holstein, Kiel, Germany; 2 Department of Translational Sciences, Research & Early Development, LEO Pharma, Ballerup, Denmark

**Keywords:** 3D skin models, Atopic dermatitis, Hair follicle–derived keratinocytes, IL-13, IL-4

## Abstract

Keratinocytes (KCs) from healthy donors stimulated with type 2 cytokines are often used to experimentally study atopic dermatitis (AD) inflammatory responses. Owing to potential intrinsic alterations, it seems favorable to use KCs from patients with AD. KCs isolated from hair follicles offer a noninvasive approach to investigate AD-derived KCs. To evaluate whether such AD-derived KCs are suitable to mimic AD inflammatory responses, we compared hair follicle–derived KCs from healthy donors with those from patients with AD in a type 2 cytokine environment. Stimulation of AD-derived KCs with IL-4 and IL-13 induced higher expression changes of AD-associated markers than that of healthy KCs. The combination of IL-4 and IL-13 generally induced highest expression changes, but IL-13 alone also induced significant changes of AD-specific markers. Similar to the 2-dimensional cultures, IL-4/IL-13 stimulation of 3-dimensional skin models generated with AD-derived KCs modulated the expression of several AD-relevant factors. Whole-transcriptome analysis revealed that IL-4 and IL-13 acted similarly on these 3-dimensional skin models. Histologically, IL-13 alone and in combination with IL-4 increased epidermal spongiosis, a histological hallmark of AD skin. Taken together, our pilot study suggests that hair follicle–derived KCs from patients with AD represent a useful model system to study AD-related inflammation in a personalized in vitro model.

## Introduction

Atopic dermatitis (AD) is a chronic inflammatory skin disease characterized by a disturbed skin barrier, type 2–dominated inflammation and microbial dysbiosis in favor of members of the genus *Staphylococcus*, particularly *Staphylococcus aureus* (Langan et al, 2020; Ständer, 2021). Although many studies have provided deep insight into the underlying dysregulated biological mechanisms, the pathogenesis of this disease is still not fully understood. Accordingly, established therapy approaches are symptomatically oriented using anti-inflammatory and anti-infective strategies. The use of novel specific target-directed biologics may offer promising new therapy options. A novel class of biologics to treat AD is based on antibodies targeting the type 2 cytokines IL-4 and IL-13 (Stölzl et al, 2021).

IL-4 signaling is initiated through 2 types of heterodimeric transmembrane receptor complexes: the type I receptor, which is primarily expressed on hematopoietic immune cells, exclusively binds IL-4, and is comprised of IL-4RA and a common γ chain; the type II receptor, which binds both IL-4 and IL-13, is comprised of IL-4RA and IL-13RA1 subunits and is expressed on many cell types, including epithelial cells (McCormick and Heller, 2015). IL-13 but not IL-4 can additionally bind a unique receptor chain referred to as the IL-13RA2. Because the intracellular domain of IL-13RA2 is believed to be too short to initiate downstream signaling, it is mainly considered as a decoy receptor to dampen activity of IL-13 (Furue et al, 2020). However, studies with human cancer cells and some mice studies suggest that IL-13RA2 can activate the activator protein-1 signaling pathway and serve as a signaling receptor for IL-13 (Fichtner-Feigl et al, 2006; Fujisawa et al, 2021; Gour and Wills-Karp, 2015; McCormick and Heller, 2015). The involvement of different receptors may contribute to the different functions exerted by IL-4 and IL-13 (McCormick and Heller, 2015). Interestingly, IL-4 and IL-13 induce the expression of IL-13RA2 but not of IL-4RA and IL-13RA1 (David et al, 2001), and overexpression of IL-13RA2 in AD has been reported (Tsoi et al, 2019; Xiao et al, 2021).

Because keratinocytes (KCs) in AD may have intrinsic alterations (Rodríguez et al, 2014; Sahlén et al, 2021), it seems favorable to directly use AD-derived KCs in experimental settings/studies. In this context, isolation and propagation of hair follicle–derived KCs avoids the use of invasive surgical methods for isolation of KCs. Such KCs have been successfully isolated from plucked hair follicles (Kopfnagel et al, 2011; Löwa et al, 2018; Matsuzawa et al, 2019; Nakano et al, 2016). In this study, we report the generation of 2-dimensional and 3-dimensional (3D) in vitro models based on AD hair follicle–derived KCs as a useful model system to study the effects of IL-4 and IL-13 on AD KCs.

## Results

### Establishment of a protocol for the isolation and cultivation of KCs derived from hair follicles

**Figure 1:**
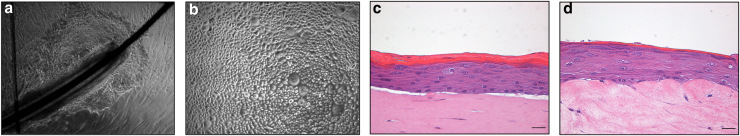
**Overview of the isolation and cultivation process of hair follicle–derived keratinocytes and resulting 3D skin model.** (**a**) Plucked hair from patients with AD or healthy donors with outer root sheets was embedded in fibroblasts; after 7–21 days, keratinocytes grew out. (**b**) Hair follicle–derived keratinocytes were propagated and stimulated as monolayer cells. Hair follicle–derived keratinocytes were used to generate a 3D skin model from (**c**) patients with AD or (**d**) healthy donors. Bars = 50 μm. 3D, 3-dimensional; AD, atopic dermatitis.

To study KCs derived from patients with AD without resorting to invasive sampling such as punch biopsies, we established a protocol for isolation of KCs from hair follicles. Using an optimized protocol for both the isolation and cultivation of hair follicle–derived KCs, we were able to successfully isolate and cultivate KCs from healthy individuals and patients with AD (Figure 1).

### Differential effects of IL-4 and IL-13 on KCs derived from healthy subjects and patients with AD

**Figure 2:**
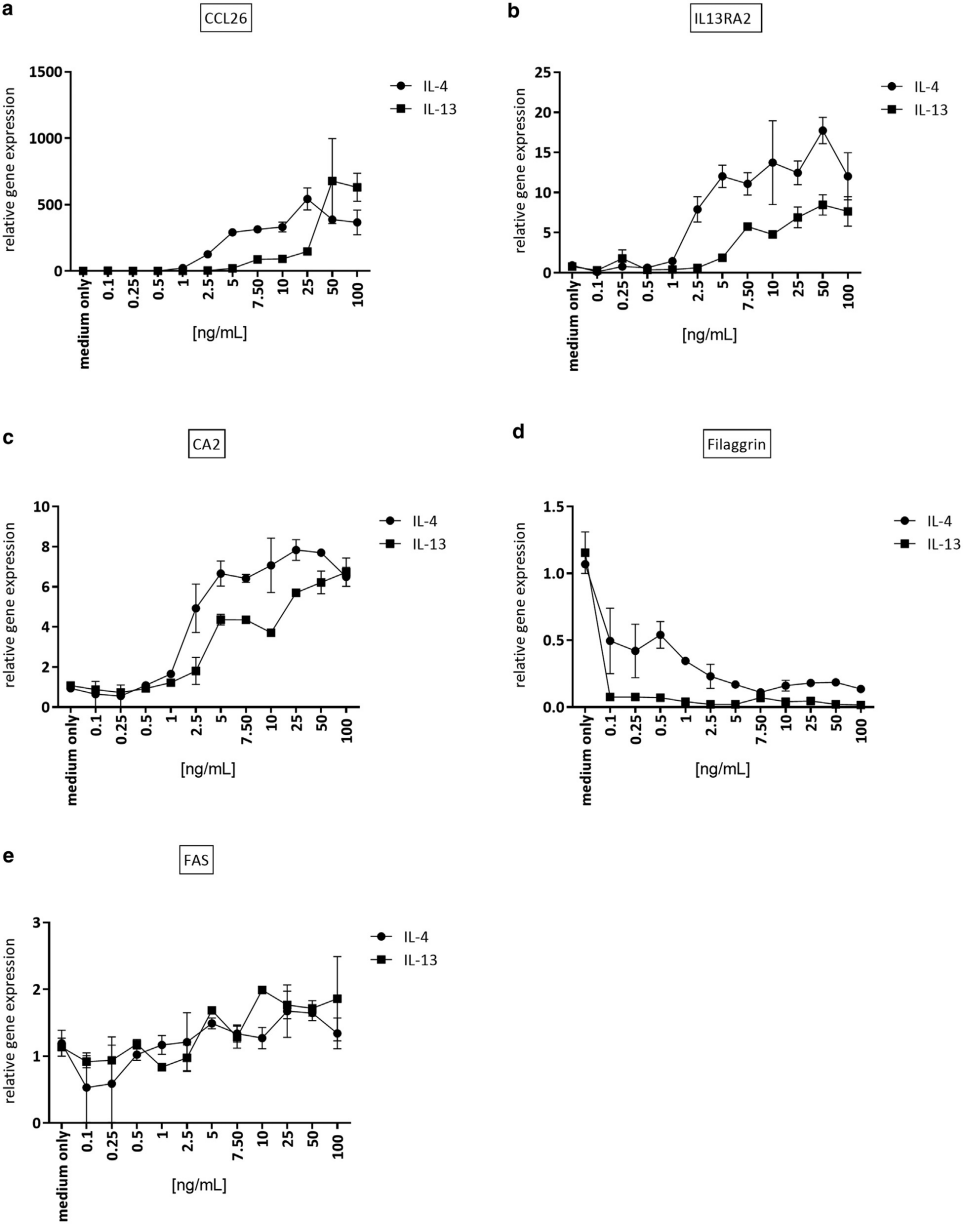
**Dose–response of IL-4– and IL-13–treated hair follicle–derived AD keratinocytes.** Keratinocytes isolated from hair follicles from a patient with AD (FDK-AD) were stimulated in 2D cultures with different concentrations of IL-4 and IL-13 for 24 hours. Gene expressions of (**a**) *CCL26*, (**b**) *IL13RA2*, (**c**) *CA2*, (**d**) *FLG*, and (**e**) *FAS* were analyzed by RT-qPCR. Bars are the mean ± SEM of 2 separate stimulations of 1 experiment. 2D, 2-dimensional; AD, atopic dermatitis; FDK-AD, follicle-derived keratinocyte from atopic dermatitis.

**Figure 3:**
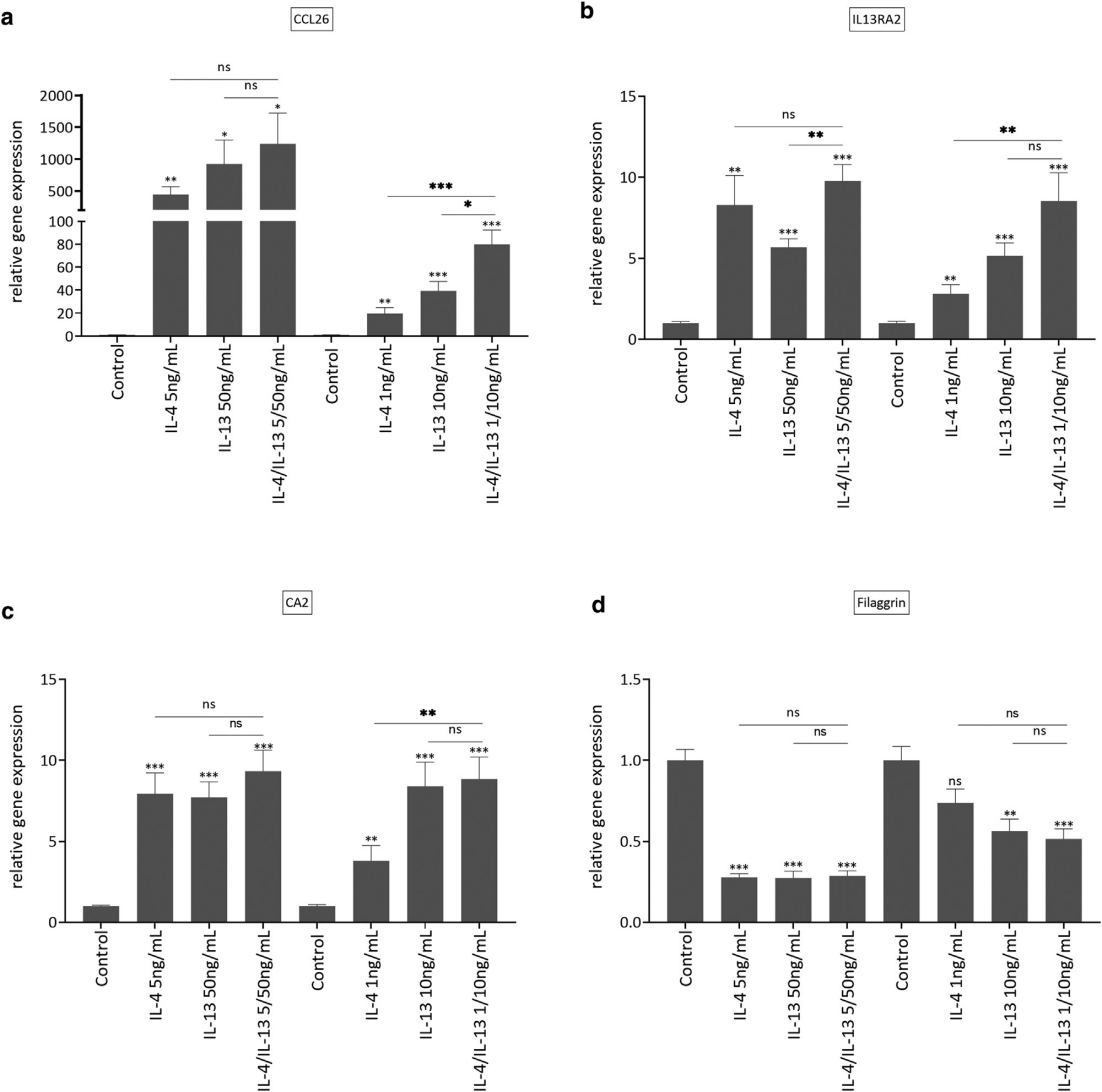
**Comparison of the effects of different IL-4 and IL-13 concentrations alone or in combination (ratio of 1:10) on hair follicle–derived AD keratinocytes.** Keratinocytes isolated from hair follicles from 3 different AD donors (FDK-AD) were stimulated in 2D cultures for 24 h either with 5 ng/ml IL-4 and 50 ng/ml IL-13 or 1 ng/ml IL-4 and 10 ng/ml IL-13 separately or with both cytokines in combination. Gene expressions of AD markers (**a**) *CCL26*, (**b**) *IL13RA2*, (**c**) *CA2*, and (**d**) *FLG* were analyzed by RT-qPCR. Bars are the mean ± SEM of cumulative data of triplicate stimulations from 3 independent experiments (∗*P* < .05, ∗∗*P* < .01, and ∗∗∗*P* < .001, with ANOVA and Holm–Sidak’s multiple comparisons test). 2D, 2-dimensional; AD, atopic dermatitis; FDK-AD, follicle-derived keratinocyte from atopic dermatitis; h, hour; ns, not significant.

To analyze the role of IL-4 and IL-13 in AD, we stimulated KCs derived from hair follicles from either healthy subjects (follicle-derived KCs [FDKs]) or age- and sex-matched patients with AD (FDKs from AD [FDK-ADs]). A dose–response experiment with increasing concentrations of IL-4 or IL-13 revealed that 50 ng/ml achieved high effects in gene expression changes of *CCL26*, *IL13RA2*, *CA2*, and *FLG* (Figure 2). An RNA-sequencing (RNA-seq) analysis of full skin biopsies from patients with AD showed increased levels of IL-13 and very low expression of IL-4 (at least 10-fold lower; results derived from Möbus et al [2021]). In addition, Koppes et al (2016) measured the protein levels of IL-13 and IL-4 in tape strips from patients with AD and reported around 7-fold higher levels of IL-13 (80.9 ng/μg protein) versus IL-4 (11.4 ng/μg protein) but noticed that the levels of IL-4 were only detectable in around 45% of the AD samples. We therefore decided to stimulate KCs with a 10:1 ratio of IL-13 to IL-4. On the basis of the dose–response experiment (Figure 2), we initially used 50 ng/ml IL-13 and 5 ng/ml IL-4 alone and in combination. For comparison, we also used lower concentrations of IL-13 (10 ng/ml) and IL-4 (1 ng/ml). These experiments revealed more pronounced differences in single cytokine stimulations than in cytokine combinations when using 10 ng/ml of IL-13 and 1 ng/ml of IL-4 (Figure 3). Thus, we continued to stimulate KCs with 10 ng/ml of IL-13 and 1 ng/ml of IL-4.

**Figure 4:**
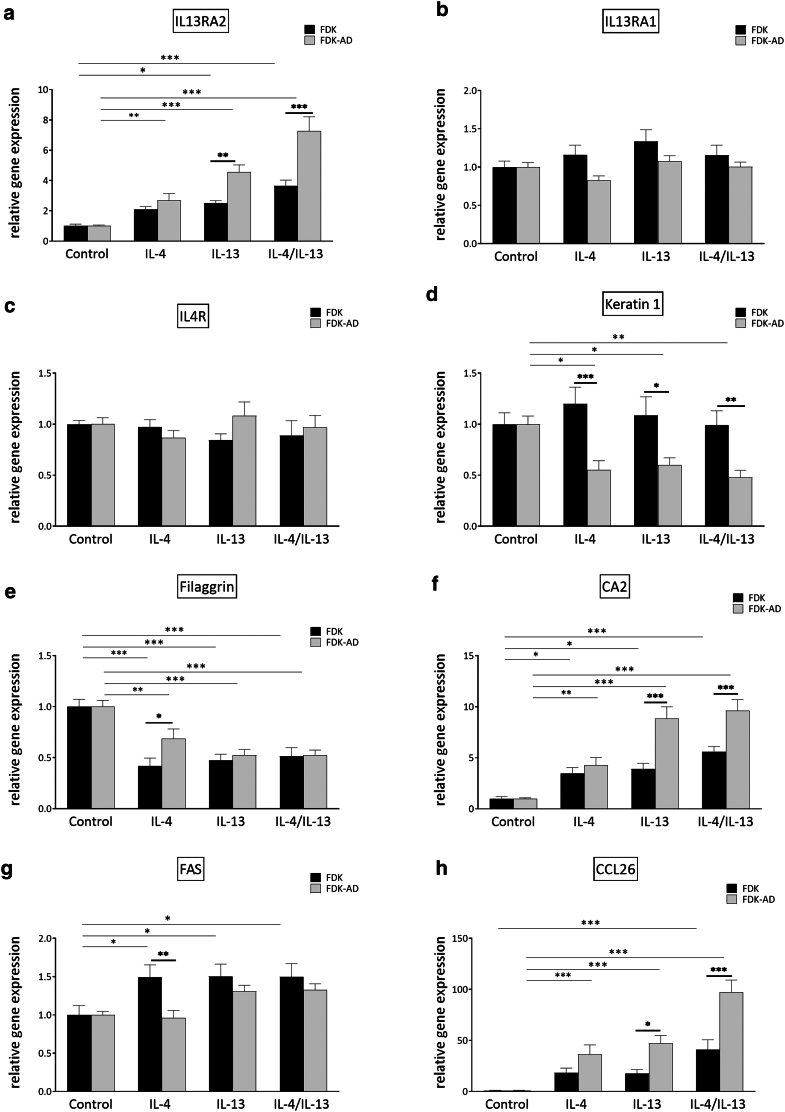
**Type 2 cytokine responses of AD-derived keratinocytes show higher expression changes of AD-relevant factors.** FDKs and FDK-ADs were cultivated as 2D monolayers in 24-well plates to a 100% confluence and stimulated with IL-13 and/or IL-4 for 24 h, respectively. (**a–h**) Different AD typical marker gene expression was analyzed by RT-qPCR. Bars are the mean ± SEM of cumulative data from 6 independent experiments, each with a different AD donor and a matched healthy control donor. In each individual experiment, 3 stimulation experiments in separate wells were performed and individually analyzed and used for statistical analysis (n = 18) (∗*P* < .05, ∗∗*P* < .01, and ∗∗∗*P* < .001, with 2-way ANOVA and Holm–Sidak’s multiple comparisons test). 2D, 2-dimensional; AD, atopic dermatitis; FDK, follicle-derived keratinocyte; FDK-AD, follicle-derived keratinocyte from atopic dermatitis; h, hour.

Stimulation of both FDK and FDK-AD showed that IL-4 and IL-13 induced changes in the expression of known AD markers associated with inflammatory responses and KC differentiation (Figure 4). In particular, IL-4 and IL-13 induced a higher expression change of some AD-associated genes such as *CCL26*, *CA2*, *IL13RA2*, and keratin 1 gene *KRT1* in FDK-AD than in FDK. In FDK-AD, IL-13 overall induced a higher effect than IL-4 at the tested concentrations. Moreover, IL-13 alone induced a similar significant response as the combination treatment, except for *IL13RA2* and *CCL26*, where additive effects were seen by the combination treatment. *IL13RA1* and *IL4R* were not induced by the type 2 cytokines (Figure 4).

### IL-4 and IL-13 regulate the expression of inflammation and KC differentiation markers in stratified 3D skin models of AD

**Figure 5:**
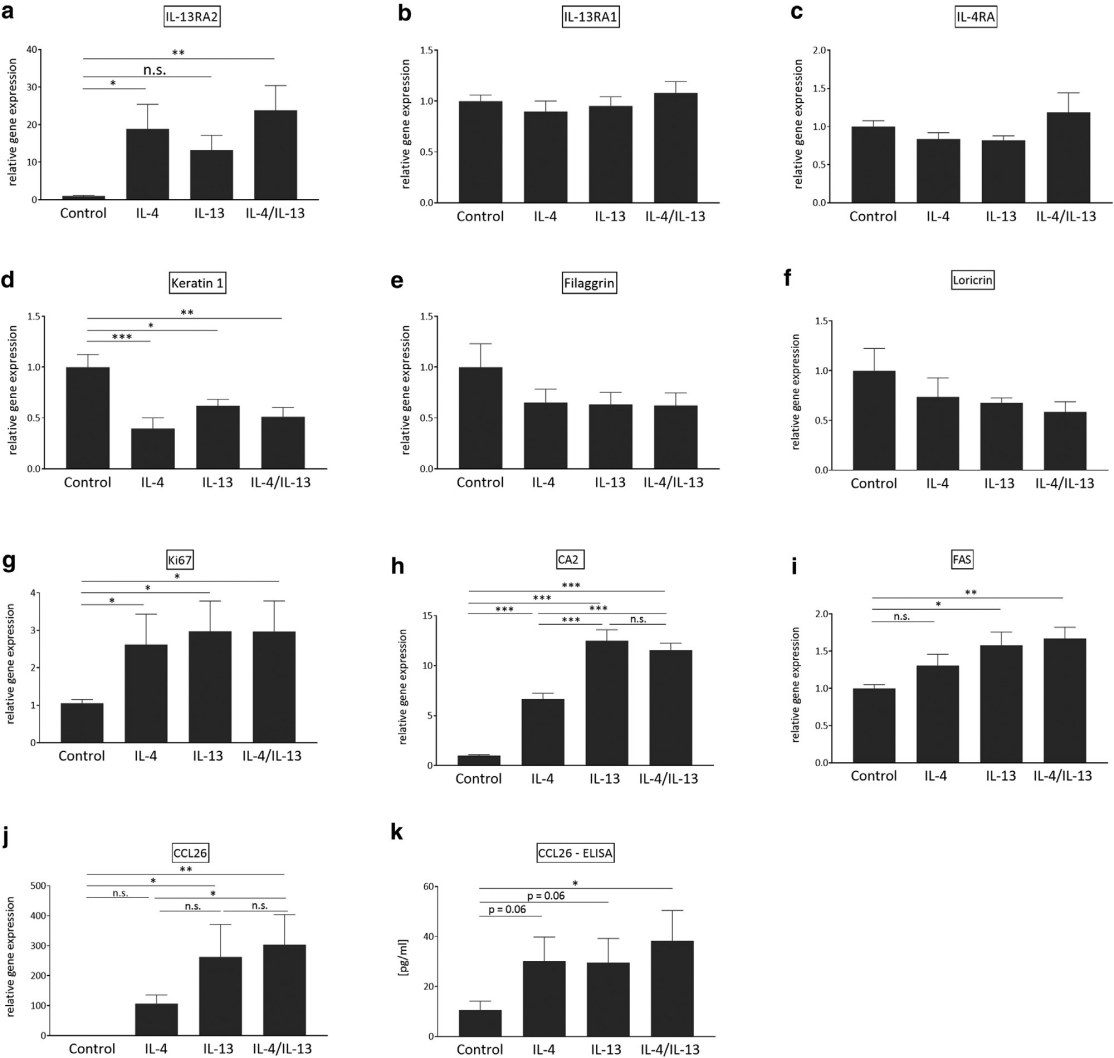
**Type 2 cytokine response of 3D skin models from AD-derived keratinocytes show typical expression patterns of AD-relevant factors.** The 3D skin models generated from AD-derived keratinocytes were stimulated for 24 h with medium alone and IL-4 (5 ng/ml) and IL-13 (50 ng/ml) alone as well as in combination. (**a–j**) Gene expression of different AD-relevant factors was analyzed by RT-qPCR. (**k**) CCL26 protein expression was determined by ELISA. Bars are the mean ± SEM of cumulative data of 10 separate 3D skin models derived from keratinocytes of 5 different donors (n = 10) (∗*P* < .05, ∗∗*P* < .01, and ∗∗∗*P* < .001, with ANOVA and Holm–Sidak’s multiple comparisons test). 3D, 3-dimensional; AD, atopic dermatitis; h, hour; ns, not significant.

To investigate the role of IL-4 and IL-13 in fully differentiated skin, we generated 3D skin models with FDK-AD according to a modified protocol of Mildner et al (2006) and Stange (2022). These skin equivalents displayed the typical structure of various layers of differentiated epidermis, including a formed stratum corneum (Figure 1). We stimulated these skin models with IL-4 and IL-13 alone and in combination in a 1:10 ratio. In this study, 5 ng/ml of IL-4 and 50 ng/ml of IL-13 were used because a higher concentration proved to be a more potent stimulus in stratified epidermis. As seen for the FDK-AD monolayer cultures, IL-4 and IL-13 alone and in combination induced an upregulation of AD-associated genes such as *CCL26*, *CA2*, and *IL13RA2* (Figure 5). Gene expression of the proliferation marker Ki-67 was also increased by IL-4 and IL-13. The effects of type 2 cytokine stimulation were also seen at protein level as demonstrated by increased release of CCL26 (eotaxin-3) (Figure 5k).

**Figure 6:**
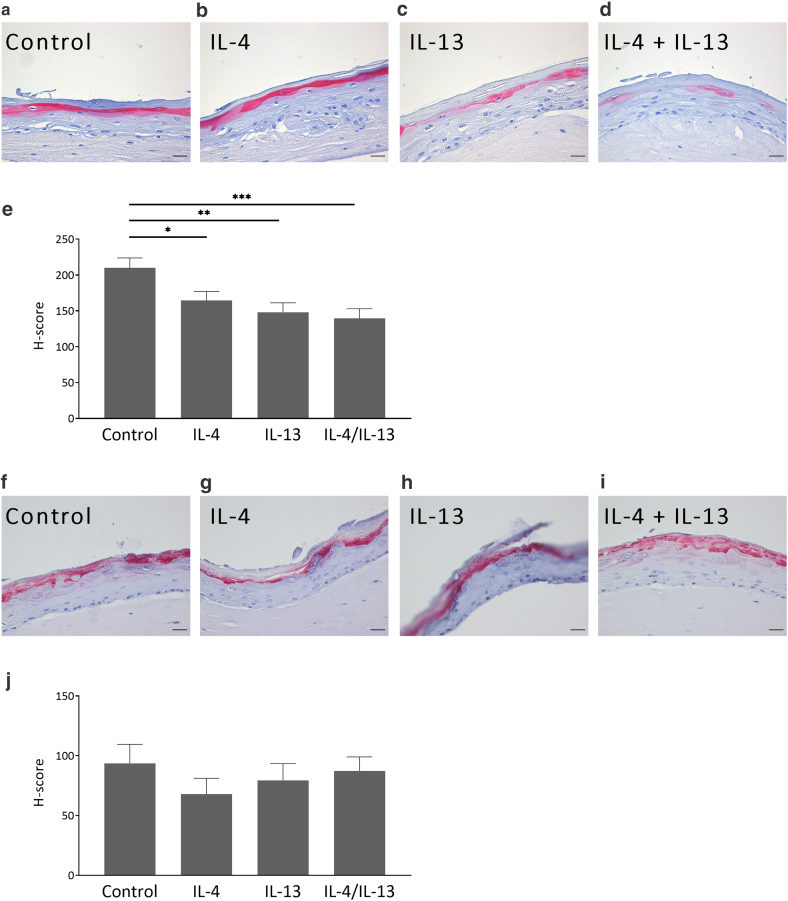
**Type 2 cytokines decrease loricrin protein expression.** The 3D skin models generated from AD-derived keratinocytes were stimulated for 24 h with (**a, f**) medium only and (**b, g**) IL-4 (5 ng/ml) and (**c, h**) IL-13 (50 ng/ml) alone and (**d, i**) in combination. (**a–d**) Loricrin and (**f–i**) filaggrin protein expression was visualized by immunohistochemistry with a specific loricrin and filaggrin antibody, respectively. H-score (Supplementary Materials and Methods) for expression of (**e)** loricrin and (**j**) filaggrin was determined by 4 laboratory assistants that scored 4 individual 3D skin models derived from 4 different donors. Bars are the mean scores of 4 individual 3D skin models ± SEM (n = 4) (bars = 50 μm) (∗*P* < .05, ∗∗*P* < .01, and ∗∗∗*P* < .001, with ANOVA and Holm–Sidak’s multiple comparisons test). AD, atopic dermatitis; h, hour.

In contrast to the induction of typical inflammation-associated genes, IL-4 and IL-13 alone and in combination significantly reduced the expression of the early differentiation marker keratin 1 (Figure 5d). IL-4/IL-13–mediated decrease in gene expression of the late differentiation markers filaggrin and loricrin did not reach statistical significance, although a clear trend of reduction was visible (Figure 5e and f). However, a significant reduction of loricrin protein expression was observed by immunostaining of 3D skin models treated with IL-4 and IL-13, whereas filaggrin downregulation reached no statistical significance (Figure 6).

**Figure 7:**
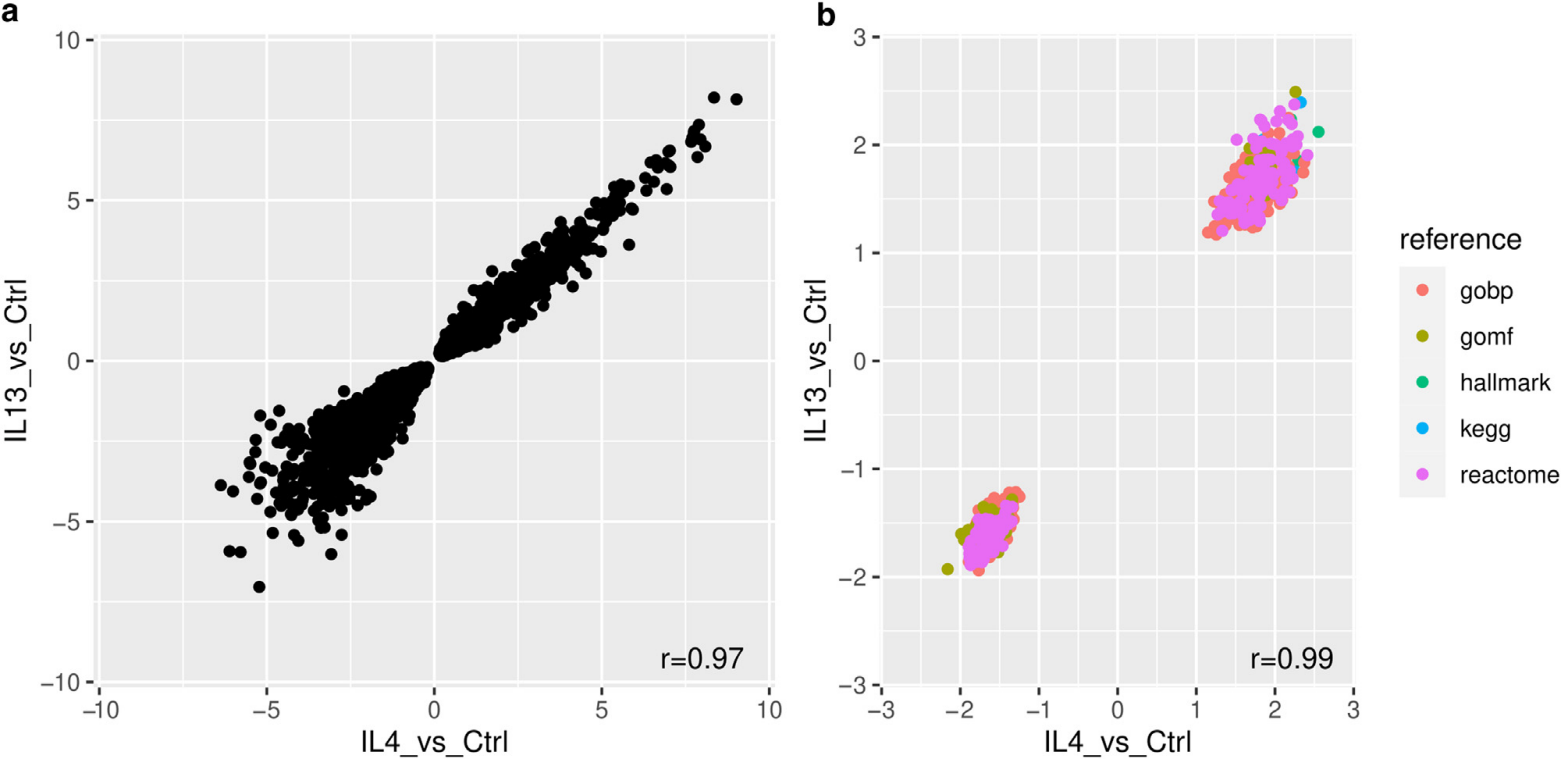
**Whole-transcriptome analysis revealed a similar gene expression pattern for IL-4 and IL-13 stimulation.** Four 3D skin models generated from AD-derived keratinocytes from 2 donors were stimulated with IL-4 (5 ng/ml) and IL-13 (50 ng/ml), and gene expression of each skin model was individually analyzed by whole-transcriptome analysis. Scatter plots are used to illustrate (**a**) concordance of gene expression (in terms of log_2_FC with differential expression *P* < .05) and (**b**) gene set enrichment (in terms of NES with enrichment *P* < .05) between IL-4 and control (denoted as IL4_vs_Ctrl) and IL-13 and control (denoted as IL13_vs_Ctrl). NES values are labeled by color in accordance with the MsigDB reference used for fgsea-based enrichment analysis (MsigDB gene set collections hallmark, kegg, gobp, gomf, and reactome). Concordance of gene expression and gene set enrichment is quantified using Pearson's correlation coefficient (r). 3D, 3-dimensional; AD, atopic dermatitis; Ctrl, control; FC, fold change; MsigDB, Molecular signature Database; NES, normalized enrichment score.

To get a more comprehensive overview of genes regulated by IL-4/IL-13, we performed whole-transcriptome sequencing analysis (RNA-seq) of the FDK-AD–derived 3D skin models stimulated with IL-4, IL-13, and IL-4 + IL-13. The results are provided in Supplementary Data S1. Genes analyzed in Figure 2 by RT-qPCR revealed a similar regulation in the RNA-seq experiment. This confirms the reliability of the RNA-seq data to get a first quantitative overview of the influence of IL-4/IL-13 on epidermal gene expression in the 3D skin model. The RNA-seq analysis indicates that most genes are similarly regulated by IL-4 and IL-13. Of note, for all significantly differentially expressed genes, the direction of regulation coincides between stimulation with IL-4 and IL-13. No gene was significantly induced by IL-4 and downregulated by IL-13 or vice versa. Accordingly, a scatter plot that correlates expression changes of IL-4 versus control with IL-13 versus control revealed a high concordance (Pearson r = 0.97) (Figure 7a). Similarly, a gene set enrichment analysis revealed high concordance on the pathway level (Pearson r = 0.99) (Figure 7b and Supplementary Data S2). In line with these data, a significant differential expression between IL-4 and IL-13 treatment of at least 2 fold was only detected for 5 genes (*S100A12*, *LINC01215*, *NKX1-2*, *MMADHC-DT*, *TRNP1*) (Supplementary Data S3). Synergistic effects of IL-4 and IL-13 treatment, respectively, were observed for only 12 genes (*NRN1*, *CLDN11*, *TMEM176B*, *FAM180A*, *ISLR*, *IL24*, *CSF2*, *ACTA2*, *TSPAN11*, *PLPPR4*, *ZP4*, *TMEM176A*) (Supplementary Data S4).

### Stimulation with IL-4 and IL-13 induces spongiosis in personalized AD 3D skin models

**Figure 8:**
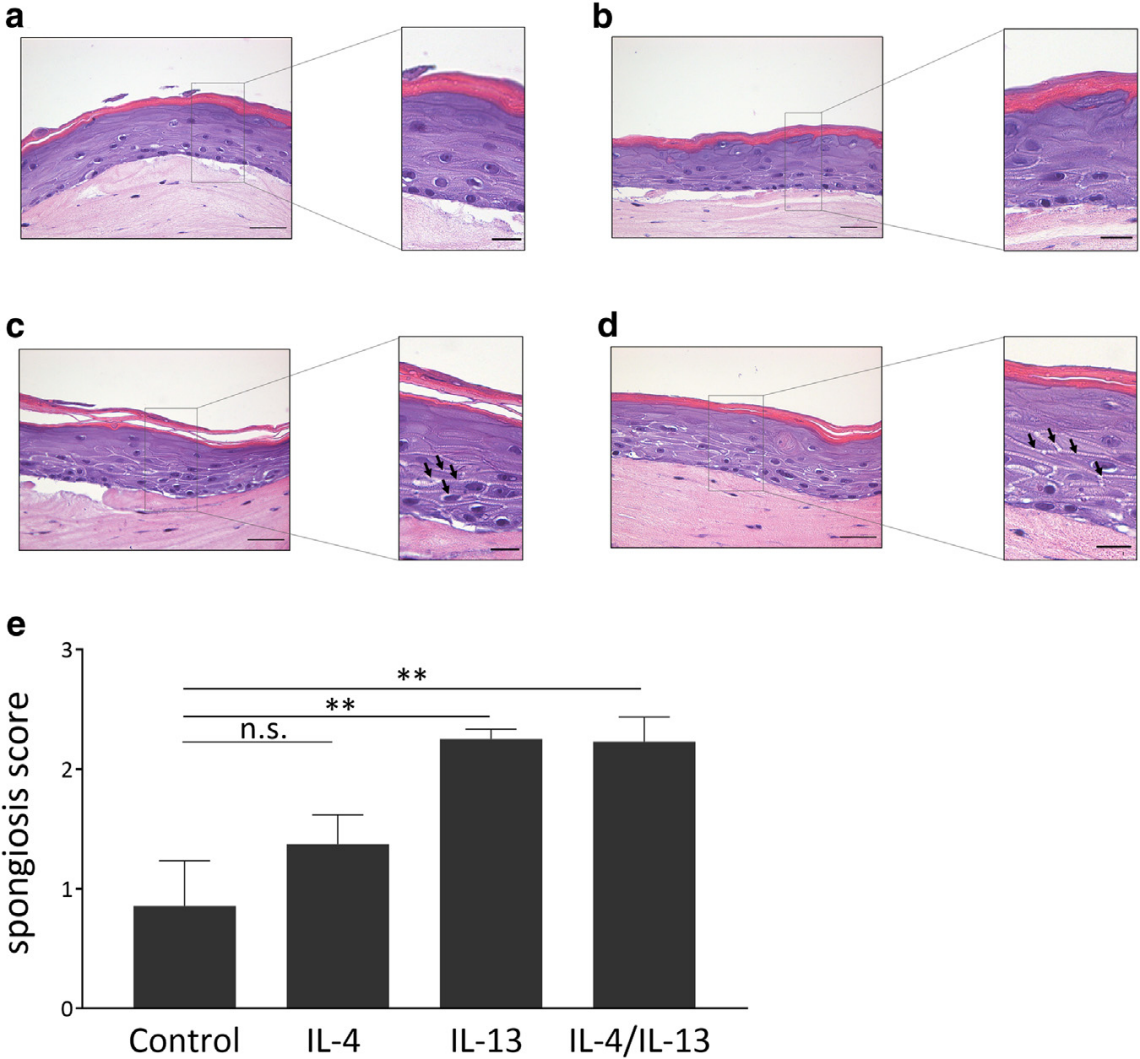
**Type 2 cytokines induce spongiosis.** The 3D skin models generated from AD-derived keratinocytes were stimulated for 24 h with (**a**) medium only and (**b**) IL-4 (5 ng/ml) and (**c**) IL-13 (50 ng/ml) alone and (**d**) in combination. (**c**) H&E staining: stimulation with IL-13 reveals the presence of spongiosis-like widened intercellular spaces between the keratinocytes, suggesting a disruption of desmosomes. This effect was enhanced by (**d**) IL-13 + IL-4 but was not seen with (**b**) IL-4 only. (**e**) Subjective scoring of the H&E-stained slides by 6 independent researchers from 4 stimulated 3D skin models derived from 4 different donors reinforced these effects significantly. Bars are the mean scores of 4 individual 3D skin models ± SEM (n = 4) (bars = 50 μm; bars in enlarged sections = 20 μm) (∗∗*P* < .01, with ANOVA and Holm–Sidak’s multiple comparisons test). AD, atopic dermatitis; h, hour; ns, not significant.

Stimulation of 3D skin models with IL-4 plus IL-13 revealed the presence of spongiosis-like widened intercellular spaces between the KCs, suggesting a disruption of desmosomes. These structures resemble acute AD lesions that often involve spongiosis in the suprabasal epidermal layers. A similar effect was seen by IL-13 alone but less by IL-4 (Figure 8a–d). A visual scoring by microscopy revealed similar results (Figure 8e).

## Discussion

Although a lot of progress has been made in deciphering the pathogenesis of AD, the exact molecular mechanisms of this disease are still emerging (Langan et al, 2020; Ständer, 2021). The aim of this study was to analyze the effects of the major AD-related type 2 cytokines IL-4 and IL-13 on AD-derived KCs. To this end, we isolated and cultivated KCs derived from hair follicles from either healthy subjects (FDKs) or patients with AD (FDK-ADs) and stimulated these cells with IL-4 and IL-13 separately and in combination. We used 10-fold higher concentrations of IL-13 than of IL-4 because IL-13 is more abundant than IL-4 in the skin of patients with AD (Möbus et al, 2021). The highest expression changes of several AD-associated factors such as CCL26 (Owczarek et al, 2010), CA2 (Kamsteeg et al, 2007), IL-13RA2 (Furue et al, 2020), and keratin 1 (Totsuka et al, 2017) were observed in FDK-ADs under the combined influence of IL-4 and IL-13. Induction of *CCL26*, *CA2*, and *IL13RA2* by IL-4 and IL-13 in combination and by IL-13 alone was significantly higher in FDK-ADs than in healthy FDKs. Moreover, IL-4, IL-13, and the combination of IL-4 and IL-13 significantly reduced *KRT1* expression in FDK-ADs but not in healthy FDKs. This indicates that the use of AD-derived KCs to study AD-related inflammation in vitro represents a favorable option.

Similar as observed for the FDK-AD monolayer cultures, in 3D skin models using FDK-AD, IL-4 and IL-13 in combination but also IL-4 and IL-13 separately induced the gene expression of several AD-associated markers such as CCL26, CA2, and IL-13RA2. This indicates that both cytokines have the capacity to induce AD-associated factors in this skin model generated with AD-derived KCs. IL-4 and IL-13 also induced the gene expression of Ki-67, a marker for cell proliferation. This suggests that IL-4 and IL-13 have a proliferative effect on KCs that may contribute to the increased KC proliferation rate associated with AD (Gittler et al, 2012; Suárez-Fariñas et al, 2011).

Our results further revealed a reduced keratin 1 gene expression in the 3D skin models treated with IL-4 and IL-13 alone and in combination. Keratin 1 is a major constituent of the intermediate filament cytoskeleton in the suprabasal epidermal layers and is typically expressed in early differentiating KCs. Our data are in concordance with a decreased expression of keratin 1 observed in lesional AD skin (Totsuka et al, 2017) and confirm the detrimental influence of these type 2 cytokines on epidermal differentiation.

Filaggrin is an important structural protein of the terminal differentiation process and plays a major role in the physical barrier of the stratum corneum. Filaggrin has been shown to play an important role in AD, and loss-of-function alterations in the gene encoding filaggrin have been identified as a significant genetic risk factor of AD (Langan et al, 2020). Previous studies have shown that the expression of filaggrin is reduced in lesional AD skin (Furue, 2020). Although we observed a trend in downregulation of filaggrin gene and protein expression by IL-4 and IL-13, it did not reach statistical significance. This is in line with data from Hönzke et al (2016) who observed no downregulation of filaggrin by IL-4/IL-13. It is likely that an application of IL-4/IL-13 to the already fully developed skin model has less influence than the application during development of the 3D skin model, a hypothesis that remains to be investigated in further studies.

Loricrin, similar to filaggrin, is also an important barrier protein that facilitates terminal differentiation of the epidermis and formation of the skin barrier. Reduced loricrin expression has been reported in AD skin (Kim et al, 2008). Similarly as seen for filaggrin, IL-4/IL-13 showed a trend in reduction of loricrin gene expression. Immunohistological analyses revealed a significant downregulation of loricrin protein expression by IL-4 and IL-13 alone or in combination. This confirms the negative influence of IL-4/IL-13 on loricrin expression, which may contribute to barrier disruption associated with AD (Kim et al, 2008).

Our RNA-seq study verified the influence of IL-4/IL-13 on gene expression of several AD-related factors in the 3D skin model generated with FDK-AD. IL-4 and IL-13 induced genes that have been reported to be strongly upregulated in AD lesional skin such as *CCL26* (Owczarek et al, 2010), *CA2* (Kim et al, 2008), *IL13RA2* (Xiao et al, 2021), *SERPINB4* (Sivaprasad et al, 2015; Suárez-Fariñas et al, 2015), and *TNFAIP6* (Evrard et al, 2021). Gene expression of epidermal barrier and junction proteins (eg, keratin 1 [Totsuka et al, 2017], FLG [Furue, 2020], FLG2 [He et al, 2021], loricrin [Kim et al, 2008], claudin 8 [Suárez-Fariñas et al, 2015]) and proteins involved in lipid metabolism (eg, ELOVL3 [Ewald et al, 2015]) that were downregulated in the lesional skin of patients with AD were also downregulated in our 3D skin model by IL-4/IL-13. Taken together, these data suggest a crucial influence of IL-4 and/or IL-13 to modulate the expression of some specific AD-relevant factors in vivo. In contrast, several inflammatory AD biomarkers that correlate with AD severity (Renert-Yuval et al, 2021) were not induced by IL-4/IL-13 in the 3D skin model (eg, CCL22, IL-18, S100A7, matrix metalloproteinase 12). Others such as CCL17/TARC, IL-4, IL-13, IL-22, and CCL18 were at or below the detection limit in our model, which is in concordance with leucocytes as principal source of these biomarkers.

The influence of IL-4/IL-13 on the epidermal expression of specific AD-relevant factors is also reflected by a comparison of our results with RNA-seq tape strip proﬁling data of He et al (2021) who provided a table with significantly differentially expressed genes from multiple pathways in tape-stripped skin with a fold change >2. Of note, 2 of 3 of terminal differentiation and lipid metabolism genes that were differentially expressed in lesional AD skin compared with those in healthy controls (He et al, 2021) matched with the effects of IL-4/IL-13 on these genes in our study. In contrast, <20% of immune genes differentially expressed in lesional AD skin compared with those in healthy controls (He et al, 2021) matched with IL-4/IL-13–influenced genes in our study. A reason for this low congruence in immune genes may be related to the lack of immune cells in our model. In summary, these comparable analyses suggest that IL-4/IL-13 may have a crucial influence on terminal differentiation and lipid metabolism in AD in vivo. In this regard, it is interesting that a recent study identified IL-13 and an altered lipid profile in the stratum corneum of infants as good predictors of AD onset by the age of 24 months (Berdyshev et al, 2023).

Of note, our RNA-seq study revealed that IL-4 and IL-13 acted very similarly on modulation of gene expression in the 3D skin models. No gene was found to be significantly regulated by IL-4 in an opposite direction as regulated by IL-13. In line with that, a pathway enrichment analysis revealed no marked differences between IL-4– and IL-13–treated skin models. Accordingly, treatment with IL-4 or IL-13 identified only a few genes showing a differential expression of at least 2-fold or synergistic effects. In general, the observed small differences in effect size between IL-4 and IL-13 treatment may be attributed to the specific concentrations used for stimulation but do not support general differential physiological effects of IL-4 and IL-13 in the 3D model.

Our data revealed strong gene expression upregulation of the IL-13 receptor IL-13RA2 but not IL-13RA1 and IL-4RA, which is in line with findings from studies reporting an IL-4/IL-13–mediated induction of IL-13RA2 in KCs (David et al, 2001; Ulzii et al, 2019). IL-13 but not IL-4 strongly binds to IL-13RA2 (Tollenaere et al, 2023). As mentioned in the introduction, IL-13RA2 is believed to act as a decoy receptor, although a few studies suggest a potential signaling role of this receptor. Our study supports the hypothesis that IL-13RA2 does not play an important role in AD KCs to selectively activate IL-13–mediated gene expression.

Histologically, stimulation with IL-13 alone significantly increased the presence of spongiosis-like structures in the suprabasal epidermal layers, which is a typical hallmark of AD skin (Bieber, 2010), thus confirming the use of this 3D skin model as a useful model system to study AD-related morphological changes. IL-4 had no statistically significant effect on spongiosis, and its addition to IL-13 did not further increase spongiosis. These data suggest that IL-13 alone may act as a driver of epidermal spongiosis in AD. In this regard, we observed that IL-13 alone induced the expression of *FAS* (encoding the Fas receptor) in the 3D skin model. Induction of apoptosis in AD was suggested to be mediated through Fas, and Fas-mediated apoptosis of KCs has been suggested as an important pathogenic event in the formation of spongiosis (Trautmann et al, 2000). Future experiments have to evaluate the potential apoptotic effects of IL-13 in AD.

The dominant effect of IL-13 on spongiosis together with its capacity to induce inflammatory mediators in 2-dimensional and 3D AD skin models presented in this report supports its proposed major role in AD, which is considered as an IL-13–dominant disease (Furue et al, 2019; Tsoi et al, 2019). This is supported by several studies that detected an enhanced expression of IL-13 in AD skin, whereas IL-4 was absent or almost undetectable (Möbus et al, 2021; Pavel et al, 2020; Tsoi et al, 2020, 2019). In addition, expression of IL-13 has been positively correlated with AD severity (Tsoi et al, 2019; Szegedi et al, 2015). Because our data revealed mainly identical effects of IL-4 and IL-13 on gene expression in our 3D AD models, the dominance of IL-13 in AD skin may explain why biologics targeting IL-13 such as the anti–IL-13 antibodies tralokinumab and lebrikizumab successfully improve AD skin lesions (Müller et al, 2024).

Together, our results suggest that 2-dimensional and 3D in vitro models based on hair follicle–derived AD KCs represent a useful model system to experimentally study the disease mechanisms of AD. Furthermore, personalized 2-dimensional and 3D models could help to gain more insight into individual patient-related pathophysiological mechanisms and to optimize therapeutic strategies.

## Materials And Methods

### Isolation of KCs derived from hair follicles

KCs from hair follicles were isolated according to a published protocol (Bacqueville et al, 2015; Wagner et al, 2018). First, a feeder layer of neonatal human dermal fibroblasts (number C-004-5C, Invitrogen) was built. To this end, fibroblasts were grown in 6-well plates to a confluency of nearly 100% with DMEM (Biozol) and 10% fetal calf serum (Capricorn Scientific). Afterward, fibroblasts were growth inactivated with mitomycin C (2 μg/ml, Sigma-Aldrich) for 2 hours at 37 °C and 5% carbon dioxide. Subsequently, fibroblasts were washed 5 times with PBS (Gibco) and incubated additionally overnight with DMEM and 10% fetal calf serum. Next day, approximately 5–10 plucked hairs with outer root sheets were firmly pressed into the prepared feeder layer in a growth medium containing 65% DMEM, 25% keratinocyte growth medium-2 (KGM2) (Promocell) and 10% fetal calf serum supplemented with 2 nM Triiodo-L-thyronine (Sigma-Aldrich) and 1% penicillin/streptomycin (Gibco). Growth medium was replaced every 2 days until outgrowth of KCs stopped. For the cultivation of the isolated KCs, colonies were selectively trypsinized from the feeder layer and subcultured in KGM2. Isolated KCs were cultured to 80–90% confluence and then cryo frozen for further experiments, where KCs in passage 3 were used.

### Cell culture

KCs were cultured in KGM2 + supplements + calcium chloride (1.3 mM) + penicillin/streptomycin at 37 °C and 5% carbon dioxide. KCs were stimulated in 24-well plates (Corning) with the indicated concentrations of IL-4 and IL-13 (Peprotech) for the indicated stimulation times in KGM2 + 0.1% BSA without supplements and antibiotics. Controls were treated without cytokines.

### The 3D skin model

To generate an organotypic 3D skin model, cultured hair follicle–derived human KCs were seeded onto a collagen/fibroblast matrix and allowed to develop a multilayered stratified epidermis through incubation for 1 week at the air/medium interface. The organotypic 3D skin model was constructed according to a protocol of Mildner et al (2006). Briefly, a 2-ml solution of 0.4% collagen G type I from calf skin (Biochrom) in 15 mM hydrogen chloride was mixed with 250 μl Hank's Balanced Salt Solution (10x, Gibco). This solution was further mixed with 250 μl fetal calf serum (Gibco) containing 1 × 10^5^ fibroblasts/ml (cultured from human foreskin dermis), and the total mixture was applied to a cell–culture insert (3-μm pore size, BD Bioscience) integrated into a 6 deep well cell culture companion plate (BD Bio Coat, Corning). After 2 hours at 37 °C, the collagen solution was gelled, and KGM2 medium with supplements was added (13 ml to the surrounding external well and 2 ml to the insert containing the gelled collagen matrix). After overnight incubation at 37 °C and 5% carbon dioxide, ∼1.5 × 10^6^ hair follicle–derived KCs in a total volume of 2 ml KGM2 medium were seeded onto the collagen matrix. After overnight incubation, the matrix gel was detached from the insert periphery. After an additional 24-hour incubation time, KGM2 medium of the insert and surrounding external well was removed. The external well was filled with 10 ml serum-free KC defined medium consisting of KGM2 full medium (without bovine pituitary extract and epinephrine) supplemented with 1.3 mM calcium, 50 μg/ml ascorbic acid (Sigma-Aldrich), and 0.1% BSA (fatty acid free, Roth). Medium was changed every second day. After 6 days, the skin models were stimulated for 24 hours with the indicated concentrations of IL-13 and/or IL-4 diluted in KGM2 without supplements in the external area of the inserts, respectively. Stimulation was performed in a flat bottom 6-well plate. After incubation, 2 biopsies were taken from each 3D skin model using a 6-mm biopsy punch. One biopsy was embedded in paraffin for immunohistochemical analysis, and the other biopsy was used for RNA isolation. The culture supernatant was harvested for ELISA.

### RT-qPCR

Total RNA of the KCs and the epidermis of the 3D skin biopsies was isolated using the reagent Crystal RNAmagic according to the manufacturer's instruction (Biolab Products). A total of 0.5 μg of the isolated RNA was reverse transcribed to cDNA using an oligo dT primer and 12.5 units of reverse transcriptase (PrimeScript RT Reagent Kit, TaKaRa Bio). cDNA derived from each individual stimulation corresponding to 10 ng total RNA served as template in 1 RT-qPCR reaction. RT-qPCR was performed in a StepOne Real-Time PCR System (Applied Biosystem) using TB Green Premix Ex Taq II (TaKaRa Bio). The primers in Table 1 were used with an annealing temperature of 60 °C.

**Table 1:** Primer Sequences of Used Primers for Gene Expression Analyses by RT-qPCR

Gene	Forward Primer	Reverse Primer
*KRT1*	CTTCTTCAGCCCCTCAATGT	GTACCTGGTTCTGCTGCTCC
*FLG*	GGCAAATCCTGAAGAATCCAGATG	GGTAAATTCTCTTTTCTGGTAGACTC
*CCL26*	CTTCCAATACAGCCACAAGCC	GTAGTGAATATCACAGCCCGCT
*IL13RA1*	GTCCCTGGTGTTCTTCCTGA	AGTGTGGAATTGCGCTTCTT
*IL13RA2*	CACCACAAGGAATTCCAGAAACT	ATGCATGATCCAAGCCCTC
*CA2*	AACAATGGTCATGCTTTCAACG	TGTCCATCAAGTGAACCCCAG
*FAS*	TGCAGAAGATGTAGATTGTGTGATGA	GGGTCCGGGTGCAGTTTATT
*Ki67*	TGACTTCCTTCCATTCTGAAGAC	TGGGTCTGTTATTGATGAGCC
*IL4RA*	GAGTGAGTGGAGCCCCAG	GAGTGAGTGGAGCCCCAG
*LOR*	CTCTCCTCACTCACCCTTCCT	AGGTCTTCACGCAGTCCAC
*RPL38*	TCAAGGACTTCCTGCTCACA	AAAGGTATCTGCTGCATCGAA

Abbreviations: KRT1, keratin 1; FLG, filaggrin; LOR, loricrin.

A gene-specific standard curve was generated with each primer pair using serial dilutions of cDNA derived from KCs with high gene expression levels. Expression level of each gene was normalized to the expression level of the constitutively expressed housekeeping gene *RPL38*. Relative gene expression is given as the ratio of target gene expression to the expression of *RPL38*.

### Whole transcriptome sequencing (RNA-seq)

The 3D skin models were stimulated with IL-13 and/or IL-4 as described earlier. After RNA isolation using the NucleoSpin RNA Kit (Macherey-Nagel), RNA libraries were generated according to the Illumina TruseqStranded mRNA protocol with poly-A enrichment, and whole-transcriptome sequencing was performed on a NovaSeq6000 sequencer (Illumina). Raw sequencing data were processed using TrimGalore (version 0.6.6), HISAT2 (version 2.2.1) (Kim et al, 2019), SAMtools (version 1.11) (Li et al, 2009), and Rsubread (version 2.2.6) (Liao et al, 2019) and annotated according to Ensembl (release 103, GRCh38). For further details and access to the processed sequencing data, reference is made to Gene Expression Omnibus (https://www.ncbi.nlm.nih.gov/geo/, accession GSE243634). The statistical analyses of the RNA-seq experiments are described in statistical analysis.

### Immunostaining

For immunostaining, 3D skin models were fixed with formalin and embedded in paraffin. Anti-loricrin (number PA5-30583, Invitrogen) 1:100 or anti-filaggrin (number HPA030188, Sigma-Aldrich) 1:200 polyclonal rabbit antibody, respectively, diluted in Tris buffered saline/1% BSA served as primary antibody. Biotinylated swine anti-rabbit (number E0431, Dako Agilent) 1:300 diluted in Tris buffered saline/1% BSA was used as secondary antibody. Blocking was performed with Tris buffered saline/12% BSA, and the Vectastain Elite ABC complex and Vector Nova Red-Substrate (Vector Laboratories) were used for development.

**Figure 9:**
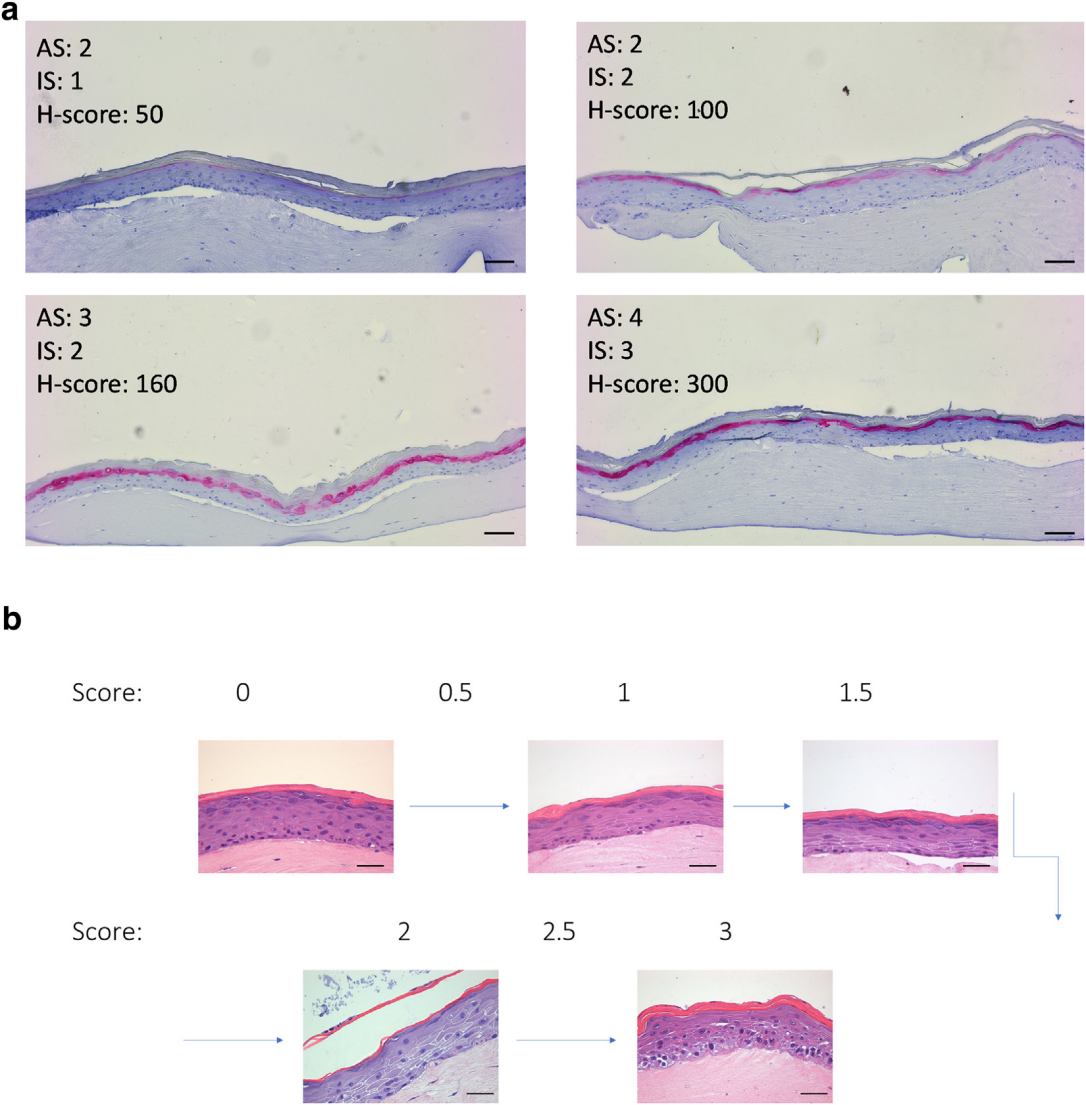
**Exemplary explanation of the histoscoring from immunohistochemistry and scoring classification of spongiosis in H&E-stained 3D skin equivalents.** (**a**) As an additional decision-making aid for the histoscorers, the representation of loricrin staining of 3D skin with assigned AS and IS served as an example (bars = 100 μm). A subdivision from 0 to 3 in steps of 0.5 was estimated for the spongiosis scoring. (**b**) As an example, a scoring division from H&E-stained 3D skin equivalents was specified for the scorers (bars = 50 μm). 3D, 3-dimensional; AS, area score; H&E, hematoxylin and eosin; IS, intensity score.

For semiquantitative analysis of the expression patterns of filaggrin and loricrin, a H-score (histoscore) analysis was performed (Numata et al, 2013). For this purpose, all stained slides (2 per individual skin model) from 4 individual 3D skin models derived from 4 different donors were examined anonymized using a Leica DM IL LED Fluo microscope (Leica Microsystems) at ×10 magnification over the entire area and scored by 4 independent laboratory assistants according to the following criteria: 0 = absent, 1 = weak, 2 = moderate, and 3 = strong staining as well as the percentage of the corresponding stained area, which was determined by an area score (Table 2 and Figure 9a [for an example]). The H-score was calculated by the sum of the staining intensities multiplied by the percentage area intensities. The H-score varies from 0 to 300.

**Table 2:** Classification of the Assessment Criteria for the H-Score Scoring of filaggrin and Loricrin Staining

Stained Area (%)	Area Score	Staining Intensity	Intensity Score
0	0	None	0
20	1	Weak	1
50	2	Moderate	2
80	3	Strong	3
100	4

### Spongiosis score

Six independent researchers received randomized and anonymized H&E-stained photographs of 4 different 3D skin models. As an indication of the scoring of spongiosis, the scorer received the examples shown in Figure 9b. After scoring, the score points were summarized, and the spongiosis score was determined using mean values.

### ELISA

Protein levels of CCL26 were detected using the Human Eotaxin-3 (CCL26) Mini ABTS ELISA Development Kit (number 900-M167, Peprotech), according to the manufacturer's instructions.

### Statistical analysis

Statistical analysis and figure drawing were done with GraphPad Prism 8.0. The normal distribution of the datasets was checked by Shapiro–Wilk test, and data were analyzed by 1-way or 2-way ANOVA with Holm–Sidak’s multiple comparisons test. All tests are 2 sided with a significance level of 0.05. The *P*-values are indicated by asterisks in the graphics: ∗*P* < .05, ∗∗*P* < .01, and ∗∗∗*P* < .001.

Differential expression of the RNA-seq experiments was assessed using paired sample analysis (design = ∼donor + treatment) using generalized linear models as implemented in DESeq2 (version 1.36.0) (Love et al, 2014). Concordance of gene expression, in terms of log_2_ fold-change, was quantified using Pearson's correlation coefficient (r). Subsequent gene set enrichment analysis was performed using the R-package fgsea (version 1.22.0, parameters: minSize = 8, maxSize = 500, eps = 0, nPermSimple = 10000), employing unfiltered log_2_ fold changes as the ranking metric and gene sets from the Molecular Sigature Database collection (Subramanian et al, 2005) as the reference (msigdbr, version 7.5.1, category = H [hallmark], subcategory = CP:KEGG [kegg], subcategory = GO:BP [gobp, Gene Ontology Biological Process], subcategory = GO:MF [gomf, Gene Ontology Molecular Function], subcategory = CP:REACTOME [reactome]). Concordance of gene set enrichment (in terms of normalized enrichment score) was quantified using Pearson correlation. All analyses were performed using R (version 4.2.30, R Core Team, 2021). Summary statistics for differential expression analyses and gene set enrichment analyses are provided as Supplementary Data S1 and S2, respectively.

## Ethics Statement

The use of plucked hair follicles was approved by the Ethical Committee of the Medical Faculty at Kiel University (reference number D 428/09). All study participants gave their written, informed consent.

## Data Availability Statement

Data underlying the results presented in this paper may be obtained from the corresponding author upon reasonable request. The sequencing data are deposited at Gene Expression Omnibus (https://www.ncbi.nlm.nih.gov/geo/, accession GSE243634).

## ORCIDs

Hila Emmert: http://orcid.org/0000-0002-7051-7350

Franziska Rademacher: http://orcid.org/0000-0002-2189-8783

Matthias Hübenthal: http://orcid.org/0000-0002-5956-3006

Regine Gläser: http://orcid.org/0000-0002-5819-777X

Hanne Norsgaard: http://orcid.org/0000-0002-5249-6701

Stephan Weidinger: http://orcid.org/0000-0003-3944-252X

Jürgen Harder: http://orcid.org/0000-0002-4075-4603

## Conflict of Interest

The study was funded by LEO Pharma. HN is an employee of LEO Pharma. HE received institutional research grants from LEO Pharma. SW received institutional research grants from Sanofi, LEO Pharma, and Pfizer and performed consultancies and/or lectures for Abbvie, Almirall, Eli Lilly, Galderma, Kymab, LEO Pharma, Pfizer, Regeneron, Sanofi, and Novartis. JH received institutional research grants from LEO Pharma and Sanofi. Matthias Hübenthal has performed consulting work for Sanofi. Franziska Rademacher and Regine Gläser declare no conflicts of interest.

## Supplementary Material

SupplementaryData S1 **Shown are the unfiltered summary statistics for differential expression analysis of 4 3D skin models generated from AD-derived keratinocytes stimulated with IL-4 (5 ng/ml) and IL-13 (50 ng/ml).** For each contrast/comparison (IL4_vs_Ctrl, IL13_vs_Ctrl, IL4_IL13_vs_Ctrl, IL4_vs_IL4_IL13, IL13_vs_IL4_IL13, and IL4_vs_IL13), gene identifiers (ENSEMBL_ID and HGNC_SYMBOL), and DESeq2 summary statistics (baseMean, FoldChange, log2FoldChange, lfcSE, stat, pvalue, and padj) are provided. For further details, see the legend sheet. 3D, 3-dimensional; AD, atopic dermatitis; Ctrl, control; DESeq2, xxx; ID, identification.

Supplementary Data S2. **Shown are the unfiltered summary statistics for gene set enrichment analysis of 4 3D skin models generated from AD-derived keratinocytes stimulated with IL-4 (5 ng/ml) and IL-13 (50 ng/ml).** For each contrast/comparison (IL4_vs_Ctrl, IL13_vs_Ctrl, IL4_IL13_vs_Ctrl, IL4_vs_IL4_IL13, IL13_vs_IL4_IL13, and IL4_vs_IL13) and each reference (MSigDB gene set collections hallmark, kegg, gobp, gomf, and reactome), pathways as well as respective fgsea summary statistics (pval, padj, log2err, ES, and NES) are reported. For further details, see the legend sheet. 3D, 3-dimensional; AD, atopic dermatitis; Ctrl, control; MSigDB, Molecular signature Database; NES, normalized enrichment score.

Supplementary Data S3 **Expression differences of 4 3D skin models generated from AD-derived keratinocytes stimulated with IL-4 (5 ng/ml) and IL-13 (50 ng/ml).** Shown are transcripts with FCs increased/decreased by at least 2 folds when comparing IL4_vs_Ctrl (FC1) with IL13_vs_Ctrl (FC2). The magnitude of expression changes is estimated irrespective of the direction. Accordingly, either FC1/FC2 (for FC1/FC2 ≥ 1) or FC2/FC1 (for FC1/FC2 < 1) is used to calculate ratios of FCs. Further details on applied filter criteria and the meaning of the individual columns are provided within the sheet differences_legend. 3D, 3-dimensional; AD, atopic dermatitis; FC, fold change.

Supplementary Data S4 **Expression synergies of 4 3D skin models generated from AD-derived keratinocytes stimulated with IL-4 (5 ng/ml) and IL-13 (50 ng/ml).** Shown are transcripts with FCs increased/decreased by at least 2 folds when comparing the combined treatment (IL4_IL13_vs_Ctrl) with respect to the individual treatments (IL4_vs_Ctrl and IL13_vs_Ctrl). The magnitude of expression changes is estimated irrespective of the direction. Accordingly, either FC (for FC ≥ 1) or 1/FC (for FC < 1) is used to calculate ratios of FCs. Further details on applied filter criteria and the meaning of the individual columns are provided within the sheet synergies_legend. 3D, 3-dimensional; AD, atopic dermatitis; Ctrl, control; FC, fold change.
