# Supplementary material for: Type 2 Cytokine–Dependent Skin Barrier Regulation in Personalized 2-Dimensional and 3-Dimensional Skin Models of Atopic Dermatitis: A Pilot Study
Source: JID Innov. 2024 Aug 30;5(1):100309. doi: 10.1016/j.xjidi.2024.100309 (PMC11460444; doi:10.1016/j.xjidi.2024.100309)
Supplement: SupplementaryData S1 — Shown are the unfiltered summary statistics for differential expression analysis of 4 3D skin models generated from AD-derived keratinocytes stimulated with IL-4 (5 ng/ml) and IL-13 (50 ng/ml). For each contrast/comparison (IL4_vs_Ctrl, IL13_vs_Ctrl, IL4_IL13_vs_Ctrl, IL4_vs_IL4_IL13, IL13_vs_IL4_IL13, and IL4_vs_IL13), gene identifiers (ENSEMBL_ID and HGNC_SYMBOL), and DESeq2 summary statistics (baseMean, FoldChange, log2FoldChange, lfcSE, stat, pvalue, and padj) are provided. For further details, see the legend sheet. 3D, 3-dimensional; AD, atopic dermatitis; Ctrl, control; DESeq2, xxx; ID, identification. [file mmc1.docx]

**Legend to supplementary Data S1**: Shown are the unfiltered summary statistics for differential expression analysis of four 3D skin models generated from AD-derived keratinocytes stimulated with IL-4 (5 ng/mL) and IL-13 (50 ng/mL). For each contrast/comparison (IL4_vs_Ctrl, IL13_vs_Ctrl, IL4_IL13_vs_Ctrl, IL4_vs_IL4_IL13, IL13_vs_IL4_IL13 and IL4_vs_IL13), gene identifiers (ENSEMBL_ID and HGNC_SYMBOL) and DESeq2 summary statistics (baseMean, FoldChange, log2FoldChange, lfcSE, stat, pvalue and padj) are provided. For further details, see the “legend” sheet.

**Legend to supplementary Data S2:** Shown are the unfiltered summary statistics for gene set enrichment analysis of four 3D skin models generated from AD-derived keratinocytes stimulated with IL-4 (5 ng/mL) and IL-13 (50 ng/mL). For each contrast/comparison (IL4_vs_Ctrl, IL13_vs_Ctrl, IL4_IL13_vs_Ctrl, IL4_vs_IL4_IL13, IL13_vs_IL4_IL13 and IL4_vs_IL13) and each reference (MSigDB gene set collections hallmark, kegg, gobp, gomf and reactome), pathways as well as respective fgsea summary statistics (pval, padj, log2err, ES and NES) are reported. For further details, see the “legend” sheet.

**Legend to supplementary Data S3:** Expression differences of four 3D skin models generated from AD-derived keratinocytes stimulated with IL-4 (5 ng/mL) and IL-13 (50 ng/mL). Shown are transcripts with fold changes (FC) increased/decreased by at least 2 folds when comparing IL4_vs_Ctrl (FC1) to IL13_vs_Ctrl (FC2). The magnitude of expression changes is estimated irrespective of the direction. Accordingly, either FC1/FC2 (for FC1/FC2>=1) or FC2/FC1 (for FC1/FC2<1) is used to calculate ratios of fold changes. Further details on applied filter criteria and the meaning of the individual columns are provided within the sheet "differences_legend".

**Legend to supplementary Data S4:** Expression synergies of four 3D skin models generated from AD-derived keratinocytes stimulated with IL-4 (5 ng/mL) and IL-13 (50 ng/mL). Shown are transcripts with fold changes (FC) increased/decreased by at least 2-folds when comparing the combined treatment (IL4_IL13_vs_Ctrl) with respect to the individual treatments (IL4_vs_Ctrl and IL13_vs_Ctrl). The magnitude of expression changes is estimated irrespective of the direction. Accordingly, either FC (for FC>=1) or 1/FC (for FC<1) is used to calculate ratios of fold changes. Further details on applied filter criteria and the meaning of the individual columns are provided within the sheet "synergies_legend".
